# Advances in the Immunobiology of Parasitic Diseases

**DOI:** 10.3390/pathogens11070811

**Published:** 2022-07-20

**Authors:** Jorge Morales-Montor, Derek M. McKay, Luis I. Terrazas

**Affiliations:** 1 Departamento de Inmunología, Instituto de Investigaciones Biomédicas, Universidad Nacional Autónoma de México, Ciudad de México 04510, Mexico; jmontor66@biomedicas.unam.mx; 2 Gastrointestinal Research Group, Inflammation Research Network, Host-Parasite Interactions Group, Calvin, Phoebe and Joan Snyder Institute for Chronic Diseases, Department of Physiology and Pharmacology, University of Calgary, Calgary, AB T2N 4N1, Canada; dmckay@ucalgary.ca; 3 Unidad de Biomedicina, Facultad de Estudios Superiores Iztacala, Universidad Nacional Autónoma de México, Tlalnepantla 54090, Mexico

Notwithstanding that most biomedical research today focuses on the pandemic caused by the SARs-CoV-2 virus, there are many unresolved diseases that are almost forgotten worldwide. Thus, when this virus is finally under control, the diseases caused by different pathogens will remain, causing the same health problems and potentially worse due to inattention in the last two–three years. Parasitic diseases are deeply rooted in human evolution and will prevail for many years. Thus, it is critical not to forget the damage they cause to the people suffering from these infections. The only way to eradicate or control such parasitic diseases is to increase our knowledge of them. Therefore, this Special Issue aims to collate recent findings achieved, despite the pandemic, by researchers interested in studying parasitic diseases. 

In this special issue of *Pathogens* entitled “Advances in the Immunobiology of Parasitic Diseases,” the reader will find some of the hottest topics in immunoparasitology emphasizing the most studied parasitic species in the world. Readers will find contributions by world leaders and have the opportunity to read up-to-date research in the field. We mainly have original research and reviews on protozoan infections, helminthic infections, and ectoparasites. Protozoan infections lead to the number of humans infected by any parasite; therefore, controlling such parasitic diseases is critically important for human health. 

It has been broadly accepted that a coordinated interaction of the nervous, immune, and endocrine systems is crucial in maintaining homeostasis in vertebrates and vital in mammals. Such neuroimmunoendocrine connections regulate the host-parasite relationship and can push the interaction towards susceptibility or protection. In this Special Issue, there are four papers focused on this important topic. There is one on Leishmaniasis, one of the world’s most widely distributed protozoan infections that cause severe damage to human health. Sánchez-García et al. [1] present an interesting research article dealing with mast cells (MCs) in *Leishmania*-infection. They found that MCs play a crucial role during infection with *Leishmania*, which is transmitted through the bite of an infected sand fly that injects saliva together with the parasite. Sand fly saliva is a complex fluid that modulates the local host immune response. In addition, hormonal factors modulate the host immune response and alter susceptibility to infections. Thus, to assess the impact of male sex hormones on the MC response to infection, the investigators orchiectomized male mice, infected them with the parasite in the presence of sand fly salivary proteins, and analyzed the response of MCs. In orchiectomized mice, MCs showed a retarded activation pattern associated with slower degranulation and weaker TNF-α, histamine, and tryptase staining in response to the infection with *Leishmania mexicana* combined with vector-salivary proteins, as compared to sham mice. Furthermore, neutrophil infiltration was lower in orchiectomized mice, and the numbers of infected macrophages and lesion sizes were smaller. Together these results suggest that during *Leishmania*-infection, male sex hormones may modulate the MC response against the parasite and salivary proteins of the sand fly vector favoring an intense inflammatory response. The absence of male sex hormones in orchiectomized mice retards the inflammatory response, enabling better infection control and slowing of disease progression.

Furthermore, Nava-Castro et al. [2] delve into the interaction of the nervous, immune, and endocrine systems during helminth infection. The spleen is a crucial organ that regulates the neuroimmunoendocrine system. The *Taenia crassiceps*-mouse model is excellent for studying the complex host–parasite relationship, particularly sex-associated susceptibility to infection. This study aimed to determine the changes in neurotransmitters, cytokines, sex steroids, and sex-steroid receptors in the spleen of cysticercus-infected male and female mice and whole parasite counts. The authors found that the parasite load was higher in female than in male mice. The levels of the neurotransmitter epinephrine were significantly decreased in infected male animals. The expression of IL-2 and IL-4 in the spleen was markedly increased in infected mice; however, the expression of Interleukin (IL)-10 and interferon (IFN)-γ decreased. Sex-associated differences between non-infected and infected mice were also observed. The data show that estradiol levels increased in infected males but decreased in females. These studies provide evidence that infection leads to changes in neuroimmunoendocrine molecules in the spleen, and these changes are dimorphic and impact the establishment, growth, and reproduction of *T. crassiceps*. Thus, these new data support the critical role of the neuroimmunoendocrine network in determining sex-associated susceptibility to this helminth parasite. 

In the case of *Toxoplasmosis,* Alonaizan et al. [3] indicate that female mice are more susceptible to *Toxoplasma gondii*-infection, as defined by higher mortality rates compared to male mice. However, whether this effect is due to an inability to control initial parasite multiplication or to the detrimental impacts on the immune system has not been determined. Therefore, studies were undertaken to assess the influence of sex on early parasite multiplication and the immune response during *T. gondii* infection and to correlate this with disease outcome. Early parasite replication was studied by applying an in vivo imaging system (IVIS) with luciferase-expressing *T. gondii*. In parallel, immunological events were analyzed by cytometric bead array to quantify key immunological mediators. The results confirmed that female mice are more susceptible to acute infection, as determined by higher mortality rates and weight loss than similarly infected males. However, conflicting with expectations, female mice harbored lower parasite burdens during acute toxoplasmosis than male mice; also, they exhibited significantly increased production of Monocyte Chemoattractant Protein-1 (MCP-1), Interferon (IFN)-γ, and Tumour Necrosis Factor (TNF)-α. MCP-1 was found to be induced by *T. gondii* in a dose-dependent manner suggesting that the observed increased levels detected in female mice were due to a host-mediated sex difference rather than to parasite load. However, MCP-1 was not affected by the physiological concentration of estrogen or testosterone, indicating that MCP-1 differences observed between the sexes in vivo are due to an unknown intermediary factor influencing MCP-1 levels. These results suggest that a more robust immune response in females than males enhances their ability to control parasite replication but increases their morbidity and mortality to toxoplasmosis. 

Another study dealing with the role of sex hormones on susceptibility to protozoan parasites is presented by Cervantes-Candelas et al. [4], who examined the role of tamoxifen in malaria. This team point out that malaria is the most lethal parasitic disease in the world. Mortality and severity of symptoms are higher in men than women, suggesting that estrogens may regulate the immune response against malaria. Tamoxifen, a selective estrogen receptor modulator used in breast cancer treatment due to its antagonistic effect on estrogen receptors α and β, was studied because of its potential therapeutic use for several parasitic diseases. However, most studies, including one on malaria, have not addressed the immunomodulatory role of tamoxifen. In this work, the authors evaluated the effect of tamoxifen on the immune response of CBA/Ca mice against *Plasmodium berghei* ANKA. This study showed for the first time that tamoxifen increased parasite load, aggravated symptoms by decreasing body temperature and body weight, and worsened anemia. Additionally, tamoxifen significantly increased the splenic index and CD4+ and NK+ cells percentages on day eight post-infection. By contrast, tamoxifen decreased both CD8+ and B220+ populations in the spleen and reduced the serum levels of IL-2, IL-6, and IL-17. These findings support the notion that tamoxifen is a potent immunomodulator in malaria-infected mice and suggest caution when administering it to malaria-infected women with breast cancer. 

It is impossible to imagine a parasitic infection without the activation of the immune response, but is such a response enough to eliminate the parasite invasion? The study of the modulation of the immunity during parasitic infections is critical to understanding the immune evasion mechanisms used by parasites and to define the main effector mechanisms to eliminate a specific parasite, which of course are not the same for all of them. Thus, a part of this Special Issue is focused on new advances in immune cell effectors and new immune players on immunomodulation in several parasitic infections. To start this section, an essential player in innate immunity was studied by Medina-Buelvas et al. [5], who assessed a role of macrophages (MΦ). They propose that this cell type plays a crucial role in developing the protective immune response against *Trypanosoma cruzi* infection. To determine the role of the MΦ subtypes M1 and M2 in developing immunity against the Mexican strain of *T. cruzi* (Ninoa strain), they analyzed the time course of the infection. They characterized the M1 and M2 subtypes in BALB/c and C57BL/6 mice. After infection, BALB/c mice developed an increased blood parasite load and cleared the parasites one week later than C57BL/6 mice. However, similar cellular infiltrates and cardiac alterations were observed in BALB/c and C57BL/6 mice. At 36 days, the *T. cruzi* infection differentially modulated the expression of immune cells, and both BALB/c and C57BL/6 mice significantly reduced CD4+ T cells. However, BALB/c mice displayed more CD8+ T cell than C57BL/6 mice in the spleen and lymph nodes. Furthermore, BALB/c mice recruited significantly more MΦ into the spleen, while C57BL/6 had similar levels to uninfected mice. The M1 MΦ ratio increased dramatically at 3–5 days post-infection (dpi) but then decreased slightly. On the contrary, M2 MΦs were low at the beginning of the infection, but the proportion of M1 and M2 MΦ at 36 dpi was similar. Notably, the MΦ subtypes M2c and M2d significantly increased the induction of tissue repair by the end of the acute phase of the infection. These results indicate that the Ninoa strain has developed strategies to modulate the immune response, with nuanced differences depending on the host’s genetic background.

Signal transducer and activator of transcription 1 (STAT1) plays a critical role in IFN-γ-mediated immune responses and resistance to protozoan and viral infections. However, its role in immunoregulation during parasitic helminth infections is not fully understood. Becerra-Diaz et al. [6] used *stat1^−/−^* mice to investigate the role of this transcription factor during infection with the cestode *T. crassiceps*, and showed that STAT1 is a central molecule favoring susceptibility to this infection. *stat1^−/−^* mice displayed lower parasite burdens at 8 weeks post-infection compared to wildtype mice. STAT1 mediated the recruitment of inflammatory monocytes and the development of alternatively activated macrophages (M2) at the site of infection. The absence of STAT1 prevented the recruitment of CD11b+Ly6ChiLy6G− monocytic cells and, therefore, their suppressive activity. This failure was associated with the defective expression of CCR2 on CD11b+Ly6ChiLy6G− cells. Importantly, CD11b+Ly6ChiLy6G− cells highly expressed PDL-1 and suppressed T-cell proliferation elicited by anti-CD3 stimulation. PDL-1+ cells were mainly absent in *stat1^−/−^* mice. Furthermore, only *stat1^+/+^* mice developed M2s at 8 weeks post-infection. However, macrophages from both *T. crassiceps*-infected wildtype and *stat1^−/−^* mice responded to IL-4 in vitro, and both groups of mice produced the Th2 cytokine IL-13. These data suggest that CD11b+CCR2+Ly6ChiLy6G−cells give rise to M2 macrophages in this infection. In summary, a lack of STAT1 resulted in impaired recruitment of CD11b+CCR2+Ly6ChiLy6G−monocytes, failure to develop M2 macrophages, and increased resistance against *T. crassiceps* infection. 

Li et al. [7] used two experimental paradigms to explore host–helminth interactions involved in the regulation of colitis and to understand if colitis affects the outcome of helminth infection. First, male BALB/c mice infected with *Hymenolepis diminuta* were challenged 4 days later with dinitrobenzene sulphonic acid (DNBS) and necropsied 3 days later. Second, mice were infected with *H. diminuta* 3 days after DNBS treatment and necropsied 11 or 14 days post-DNBS. Mice were assessed for colitic disease severity and infectivity with *H. diminuta* upon necropsy. Supporting the concept of helminth therapy, mice were protected from DNBS–colitis when infected with *H. diminuta* only 3 days previously, along with parallel increases in splenic production of Th2 cytokines. In the treatment regimen, *H. diminuta* infection produced a slight, statistically significant, enhanced recovery from DNBS. Mice regained body weight quicker, had normalized colon lengths, and showed no overt signs of disease compared to the DNBS-only mice, some of which displayed signs of mild disease at 14 days post-DNBS. Unexpectedly, colitis did not affect the hosts’ anti-worm response. The impact of inflammatory disease on helminth infection is deserving of study in various models as auto-inflammatory diseases emerge in world regions where parasitic helminths are endemic.

Aguilar-Diaz et al [8] delved into extracellular traps’ role upon infection with ticks. The authors discuss that ticks are hematophagous ectoparasites that infest a diverse number of vertebrate hosts. Tick immunobiology plays a significant role in establishing and transmitting many pathogens to their hosts. To control tick infestations, acaricide application is commonly used with severe environmental consequences and the selection of tick-resistant populations. With these drawbacks, new tick-control methods need to be developed, and the ticks’ immune system contains many potential candidates for vaccine design. Additionally, tick immunity is based on an orchestrated action of humoral and cellular immune responses. A new method to control tick infestations through the development of vaccines is proposed as well as Extracellular Traps Formation (ETosis) in ticks as a process to eliminate their natural enemies and pathogens they transmit (vectorial capacity).

Furthermore, in the field of malaria, Carlos L. Calle [9] discusses in a review article that malaria reflects not only a state of immune activation, but also a state of general immune defect or immunosuppression, of complex etiology that can last longer than the actual episode. Inhabitants of malaria-endemic regions with lifelong exposure to the parasite show an exhausted or immune-regulatory profile compared to non- or minimally exposed subjects. Several studies and experiments to identify and characterize the cause of this malaria-related immunosuppression have shown that malaria suppresses humoral and cellular responses to both homologous (Plasmodium) and heterologous antigens (e.g., vaccines). However, the underlying mechanisms or the relative involvement of different immune cells in immunosuppression during malaria is poorly understood. Moreover, the implication of the parasite during the various stages of the modulation of immunity has not been addressed in detail. There is growing evidence of a role of immune regulators and cellular components in malaria that may lead to immunosuppression that needs further research. This review summarizes the current evidence on how malaria parasites may, directly and indirectly, induce immunosuppression and investigate the potential role of specific cell types, effector molecules, and other immunoregulatory factors.

Rajeev and collaborators [10] summarise the role that enteric tuft cells have as chemosensory epithelial cells and are gaining attention in the field of host–parasite interactions [10]. These cells express a repertoire of chemosensing receptors and mediators that have the potential to detect lumen-dwelling helminth and protozoan parasites and coordinate epithelial, immune, and neuronal cell defenses against them. This review highlights the versatility of enteric tuft cells and subtypes thereof, showcasing nuances of tuft cell responses to different parasites, with a focus on helminths, reflecting the current state of the field. The role of enteric tuft cells in irritable bowel syndrome, inflammatory bowel disease, and intestinal viral infection is assessed in the context of concurrent infection with parasites. This review also presents pertinent questions to understand the enteric tuft cell and its role in enteric parasitic infections. There is much to be done to fully elucidate the response of this intriguing cell type in parasitic-infection, and there is negligible data on the biology of the human enteric tuft cell—a glaring knowledge gap that must be filled.

Finally, Yousefi et al. discuss that several parasites have evolved to survive in the human intestinal tract, and over 1 billion people worldwide, specifically in developing countries, are infected with enteric helminths [11]. *Trichuris trichiura* is one of the world’s most common intestinal parasites that causes human parasitic infections. *Trichuris muris*, an immunologically well-defined mouse model of *T. trichiura*, is extensively used to study different aspects of the innate and adaptive components of the immune system. Studies with the *T. muris* model offer insights into understanding host immunity since this parasite generates two distinct immune responses in resistant and susceptible mouse strains. Apart from immune cells, infection with *T. muris* also influences various components of the intestinal tract, especially the gut microbiota, mucus layer, epithelial cells, and smooth muscle cells. This review analyzes different immune responses generated by innate and adaptive immune components during acute and chronic *T. muris*-infection. Furthermore, they highlight the importance of utilizing the *T. muris*-model to unravel host–parasite interactions in the context of alteration of the host’s microbiota, intestinal barrier, inflammation, innate defense, and parasite infection-mediated modulation of other immune and inflammatory diseases.

We hope that our readers will find this a fascinating and enticing first-ever Special Issue devoted to Immunoparasitology that we were proud to edit.
